# Altered Leukocyte Sphingolipid Pathway in Breast Cancer

**DOI:** 10.3390/ijms18122521

**Published:** 2017-11-24

**Authors:** Larissa P. Maia, Paula S. Santos, Patrícia T. Alves, Cláudia M. Rodrigues, Thaíse G. Araújo, Yara Cristina P. Maia, Alinne Tatiane F. Câmara, Donizeti W. Santos, Luiz Ricardo Goulart

**Affiliations:** 1 Laboratory of Nanobiotechnology, Institute of Genetics and Biochemistry, Federal University of Uberlandia, Umuarama, Uberlandia, MG 38400-902, Brazil; l.pradomaia@gmail.com (L.P.M.); paulasantos.bio@gmail.com (P.S.S.); patriciaterraalves@yahoo.com.br (P.T.A.); claudiamr.bio@gmail.com (C.M.R.); thaise_araujo@yahoo.com.br (T.G.A.); yaracpmaia@gmail.com (Y.C.P.M.); alinnetatianefaria@gmail.com (A.T.F.C.); 2 Obstetric Division, Internal Medicine, University Hospital, Federal University of Uberlandia, Umuarama, Uberlandia, MG 38405-320, Brazil; donizetw@gmail.com; 3 Department of Medical Microbiology and Immunology, School of Medicine, University of California Davis, Davis, CA 95616, USA

**Keywords:** sphingosine 1-phosphate (S1P), sphingolipids, S1P receptors, breast cancer, leukocytes

## Abstract

Sphingolipid metabolism pathway is essential in membrane homeostasis, and its dysfunction has been associated with favorable tumor microenvironment, disease progression, and chemotherapy resistance. Its major components have key functions on survival and proliferation, with opposing effects. We have profiled the components of the sphingolipid pathway on leukocytes of breast cancer (BC) patients undergoing chemotherapy treatment and without, including the five sphingosine 1-phosphate (S1P) receptors, the major functional genes, and cytokines, in order to better understand the S1P signaling in the immune cells of these patients. To the best of our knowledge, this is the first characterization of the sphingolipid pathway in whole blood of BC patients. Skewed gene profiles favoring high *SPHK1* expression toward S1P production during BC development was observed, which was reversed by chemotherapy treatment, and reached similar levels to those found in healthy donors. Such levels were also correlated with high levels of TNF-α. Our data revealed an important role of the sphingolipid pathway in immune cells in BC with skewed signaling of S1P receptors, which favored cancer development even under chemotherapy, and may probably be a trigger of cancer resistance. Thus, these molecules must be considered as a target pathway for combined BC therapeutics.

## 1. Introduction

Cancer is characterized by the accumulation of genetic and cellular alterations, which generate immune response to tumor antigens [1]. Effective responses against cancer cells depend on several steps, including tumor antigens capture by dendritic cells and presentation to T cells, which are then activated. Effector T cells traffic and infiltrate in the tumor bed, recognize and bind to cancer cells, and kill them [2,3]. Although T cells have significant function in tumor antigens recognition, all immune cell types also play important roles in tumors [4]. Monocytes are recruited to tumor tissues and differentiate into tumor-associated macrophages, performing pro-tumor functions with increased production of factors that support tumor initiation, neoplastic transformation, and distant metastasis onset [5,6]. Neutrophils are also recruited and differentiate into tumor-associated neutrophils (TANs), but their functions are unclear, since TANs produce pro-inflammatory and anti-inflammatory factors [7].

Tumor cells will also lead to the production of metabolites that function as secondary signals to all immune cells. Among these, the sphingolipid metabolites and their receptors have emerged as an important signaling pathway involved in inflammation and cancer, that modulates cell differentiation, apoptosis, proliferation and angiogenesis [8,9,10]. However, the role of sphingosine 1-phosphate receptors (S1PR) and the sphingolipid pathway in breast cancer (BC) immunity has not been well characterized [11,12].

Sphingosine 1-phosphate (S1P) is a bioactive sphingolipid generated through the activation of an enzymatic cascade that originates ceramide and subsequently sphingosine through the ceramide kinase (CERK) activity. Sphingosine is then phosphorylated in S1P by two sphingosine kinases (SPHK), SPHK1 and SPHK2 [12,13,14]. S1P regulates important biological processes, both intracellular as a second messenger, and extracellular by the interaction with five G protein-coupled receptors (GPCR), S1PR1–5, whose functions differ according to the cell type [15,16]. All immune cells express S1PRs. Dendritic cells express all five receptors, whereas T cells express S1PR1 and 4, B cell express S1PR1 and 3, macrophages express S1PR1 and 2 [17], and eosinophil express S1PR1, 2, and 3 [18]. Each receptor plays a different role in each cell type, including chemotaxis, migration, cell survival, egress, tissue infiltration, and others [19].

To the best of our knowledge, there is no characterization of the sphingolipid pathway in leukocytes in breast cancer with and without chemotherapy, and we hypothesize that the sphingolipids pathway in leukocytes is being modulated by cancer and chemotherapy and may be responsible for cancer development and progression. This study is the first one that characterizes S1PRs in lymphocytes, monocytes, and granulocytes, the expression behavior of major genes involved in the sphingolipid pathway, and the cytokines profile from BC patients undergoing chemotherapy treatment (BCC) and without treatment.

## 2. Results

### 2.1. S1P Receptors Characterization in Leukocytes

**Figure 1:**
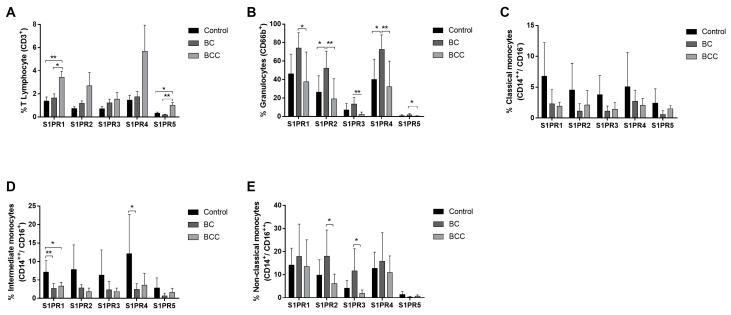
Characterization of sphingosine 1-phosphate receptors (S1PRs) in leukocytes of breast cancer patients without (BC) and with chemotherapy treatment (BCC). Expression of S1PRs was analyzed in (**A**) T lymphocytes (CD3^+^); (**B**) Granulocytes (CD66b^+^); (**C**) Classical monocytes (CD14^++^, CD16^−^); (**D**) intermediate monocytes (CD14^++^, CD16^+^) and (**E**) non-classical monocytes (CD14^+^, CD16^++^) from healthy donors (control) and BC and BCC patients. Data are expressed as means + SD. * *p* < 0.05, ** *p* < 0.01.

S1PRs were examined in blood cells from BC and BCC patients and healthy donors, with specific staining for T lymphocytes, granulocytes, and monocytes (classical, intermediate, and non-classical). Results reveled all these cells express the five receptors in a disease-independent manner (Figure 1). S1PR1 and S1PR5 were significant higher in T lymphocytes from BCC group (*p* < 0.01 and *p* < 0.05, respectively) relative to BC and control groups, although S1PR4 was the most expressed receptor in BCC. Moreover, BC also showed slightly higher expression levels of receptors than controls. Moreover, expression levels were constant in each group, except for S1PR5 (Figure 1A). Considering granulocytes, BC showed high expression of S1PRs, and both S1PR2 and S1PR4 were 2-fold higher than those in controls (*p* < 0.05) (Figure 1B). Interestingly, BCC showed lower levels in this cell type. In classical monocytes, the highest expression of receptors was observed in the control group, but with non-significant differences among groups (Figure 1C). The same profile was identified in intermediate monocytes, with 2 and 4-fold higher expression levels for S1PR1 and S1PR4 when compared to controls, respectively (*p* < 0.05) (Figure 1D). S1PRs in non-classical monocytes are more expressed in BC than in BCC, with higher surface expressions of S1PR2 and S1PR3 in BC (*p* < 0.05) (Figure 1E). In general, S1PR5 was the least expressed receptor in all cells, whereas S1PR1 and S1PR4 presented in higher levels in all immune cells analyzed.

### 2.2. Relative mRNA Expression of Major Genes of the Sphingolipid Pathway

**Figure 2:**
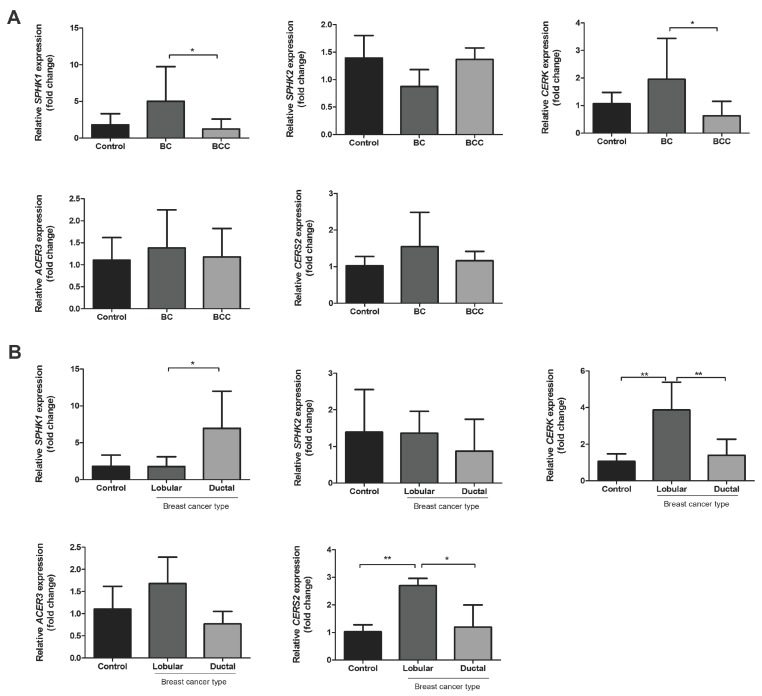
Expression levels of *SPHK1*, *SPHK2*, *CERK*, *CERS2*, and *ACER3* mRNA in blood of breast cancer patients without (BC) and with chemotherapy treatment (BCC). (**A**) Relative quantification of *SPHK1*, *SPHK2*, *CERK*, *CERS2*, and *ACER3* mRNA in whole blood of healthy donors (control) and BC and BCC patients and (**B**) after stratifying BC group in lobular and ductal. The data were normalized to *GAPDH* and calibrated with the control group. Expression levels were calculated using the 2^−^^ΔΔ*C*t^ method. The data are expressed as means + SD. * *p* < 0.05 and ** *p* < 0.01.

The relative mRNA expression of five major genes of the sphingolipid pathway was performed in peripheral blood from BC and BCC patients. The analysis revealed that all groups exhibited variable expression levels of *SPHK1*, *SPHK2*, *CERK*, ceramidase 3 (*ACER3*) and ceramide synthase 2 (*CERS2*) (Figure 2). For BC samples, the relative *SPHK1* and *CERK* mRNA expression levels were 3.8-fold and 1.3-fold higher than in BCC (*p* < 0.05), respectively. Interestingly, a comparison of the levels of *SPHK1* gene revealed that the transcript expression in invasive lobular carcinoma (ILC) was 8.0-fold higher (*p* < 0.05) than in invasive ductal carcinoma (IDC) within the BC group. Furthermore, *SPHK2*, *CERK*, *CERS2*, and *ACER3* levels were 0.5, 2.5 (*p* < 0.01), 1.5 (*p* < 0.05), and 6.9-fold higher in ILC than IDC, respectively (Figure 2B).

### 2.3. Cytokine Production

**Figure 3:**
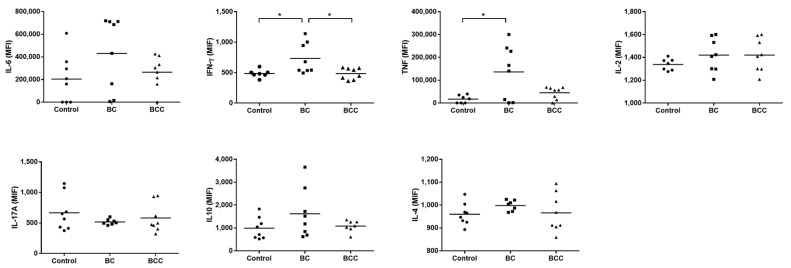
Cytokine analysis in blood of breast cancer patients without (BC) and with chemotherapy treatment (BCC). Interleukin (IL)-2, IL-4, IL-6, IL-10, IL-17A, tumor necrosis factor alpha (TNF-α) and interferon gamma (IFN-γ) levels were measured by Cytometric bead array (CBA) Human Th1/Th2/Th17 Kit in whole blood of healthy donors (control) and BC and BCC patients. Presented results are expressed in Median Fluorescence Intensity (MFI). * *p* < 0.05.

Cytokines levels were assessed by CBA in blood from BC and BCC patients. The same conditions were used for control samples. All groups expressed the seven cytokines analyzed (Interleukin (IL)-2, IL-4, IL-6, IL-10, IL-17A, tumor necrosis factor alpha (TNF-α) and interferon gamma (IFN-γ)). TNF-α and IFN-γ cytokine levels were significant higher in BC than control group (*p* < 0.05) (Figure 3). Correlation analysis was used to identify the relationship between the *SPHK1* expression and cytokines levels, and results indicated a positive correlation between *SPHK1* and TNF-α in breast cancer, *r* = 0.5287, *p* = 0.0352 (Table 1).

## 3. Discussion

We have investigated the sphingolipid pathway on breast cancer immune response through gene expression analyses of the major functional genes, all five S1PRs, and cytokines secretion, especially characterizing the involvement of the S1P signaling in the immunological cells of cancer patients. This is the first characterization of the sphingolipid pathway profile in whole blood from breast cancer patients with and without chemotherapy. We have observed a skewed profile of genes that favored high *SPHK1* expression towards the production of S1P in the blood during BC development. Such levels also reinforced the high levels of pro-inflammatory cytokines release. However, after chemotherapy treatment (BCC) patients presented diminished expression levels. We have also characterized all five S1PRs in lymphocytes, granulocytes and monocytes from both BC and BCC patients. Although cells expressed all five receptors, they varied in their surface expression, and differential expression seems to play major roles in the tumor immune system.

The characterization of all five S1PRs in lymphocytes, granulocytes, and monocytes of BC and BCC patients demonstrated that all cell types express the five receptors in both groups, in contrast with a study that has been recently reported elsewhere [19]. Indeed, S1PRs can play different functions on immune cells [17]. Decreased S1PR1 levels in BC T-lymphocytes related to BCC may be due to receptor internalization thought high concentrations of S1P in the blood [20,21], corroborated by the high *SPHK1* expression levels in BC blood cells, which may lead to exacerbated production of S1P. Furthermore, S1PR4 seems to have no influence on lymphocytes migration, but suppresses T cells proliferation [22]. This receptor was the most expressed in lymphocytes of BCC patients among all receptors, which is interestingly because chemotherapy promotes lymphopenia, causing lymphocyte depletion through apoptosis [23,24]. It is likely that high expression of S1PR4 in BCC lymphocytes may be capturing restricted levels of circulating S1P, evidenced by decreased levels of *SPHK1*, probably inhibiting other S1PRs, and attenuating lymphocyte function. It has been shown elsewhere that the S1PR4 receptor also acts by inducing a pro-inflammatory response and migration from blood into tissue in neutrophil [25], which explains the higher S1PR4 levels observed in this study, and the pro-inflammatory profile in BC samples.

Interestingly, we have found that non-classical monocytes in BC increased levels of S1PR2 and S1PR3, a profile that is abrogated under BCC. These results seem to be controversial, since S1PR2 is associated with inhibition of chemotaxis [26], whereas S1PR3 promotes chemotaxis during inflammation [27]. However, the activation of such receptors during BC may be implicated in monocyte dysfunction. High levels of S1PR1 and S1PR4 were observed in intermediate monocytes of healthy donors, despite no function has been described for these receptors in this cell type. In general, all S1PRs were highly active in classical and intermediate monocytes in healthy individuals, and depleted during BC and BCC, suggesting that these monocytes are chronically deactivated in cancer. However, the receptors expression in non-classical monocytes, which migrate to tissue and differentiate into dendritic cells and macrophages, present increased and abnormal levels in BC. Actually, information concerning the function of S1PRs in immune cells, particularly in cancer, is currently lacking.

Increased mRNA levels of *SPHK1* observed in BC blood cells are comparable with profiles found in BC tissue, associated with a poor prognostic as reported elsewhere [28,29]. Several proteins that interact with SPHK1 in MCF-7, a breast cancer cell line, were identified demonstrating their involvement with cell migration, adhesion and cytoskeletal remodeling [30]. SPHK1 is also associated with malignant transformation and cancer proliferation, among other functions that are linked to cell survival [31,32], which are corroborated by findings in MCF-7 cells [33]. Similarly, decreased levels of SPHK1 under chemotherapy were also observed in prostate cancer cell lines, which was further corroborated by in vivo studies with mice that showed SPHK1 inhibition, smaller tumor volume, and reduced occurrence and number of metastases [34]. Increased levels of *CERK* were also observed in whole blood of BC patients. CERK phosphorylates ceramide to generate ceramide 1-phosphate (C1P), a lipid that acts similarly to S1P, and particularly plays roles in cell proliferation, migration, and survival [35,36,37]. Interestingly, the gene expression profile from the control group was similar to BCC, showing that chemotherapy has effectively modulated the sphingolipids pathway, clearly demonstrating the importance of this pathway in cancer.

In the present study, the cytokine analysis showed expression of both pro- and anti-inflammatory cytokines. These results can be explained by the immune status, since simultaneous immune stimulation and immunosuppression are expressed in cancer patients without treatment [38] as well as in some patients under chemotherapy with cyclophosphamide that also present immunosuppression [39]. We have significantly observed higher levels of TNF-α in BC, which act in tumor cell survival through activation of the Factor nuclear kappa B (NF-κB) and Signal transducer and activator of transcription 3 (STAT3) signaling pathways [40]. Moreover, we showed an association between SPHK1 and TNF-α blood levels in BC. It is known that TNF-α can activate SPHK1 in endothelial cells promoting anti-apoptotic effect [41]; thus, TNF-α may be a trigger of the S1P pathway, mainly by activating SPHK1, favoring the cancer development. We have also observed high levels IFN-γ in BC patients, which may act either as tumor suppressor [42] or associated with induction of estrogen receptor [43], corroborating with the dual role of this pleotropic cytokine in tumor immune responses. BCC did not show differences in cytokines levels, possibly due to the alkylating agent cyclophosphamide that induce tumor cell death through apoptosis, and also because of it is non-immunogenicity [44].

The sphingolipid pathway has not been well characterized in blood cells yet. But, considering that platelets and erythrocytes present the highest activity of SPHK1 and S1P released in the blood [45], which is further corroborated by our data that showed increased levels of TNF-α that also correlated with high levels of SPHK1 in the blood, as also shown elsewhere [41]. It is expected that S1P may further activate S1PRs of leukocytes both systemically and locally in the tumor microenvironment, playing an important role in cancer development and progression, probably leading to tumor infiltration and/or therapy resistance. It is also important to emphasize that leukocytes are blood cells that present the expression of the full sphingolipid pathway [46]. Therefore, our data suggest that these cells are also producing their own S1P, which may be signaling either by autocrine and paracrine manner. Therefore, this is the first description of S1PRs in leukocytes with significant differences among BC patients without and with chemotherapy (BCC), which opens new avenues for investigation of drug targets, mainly by observing the variation of S1PR1 and S1PR4.

Several analogues and inhibitors of the sphingolipid metabolism have been implicated in targeted therapy [47,48,49]. Ceramide analogues induce apoptosis in human cancer cells [50,51], and the inhibitor of acid ceramidase, an enzyme that promotes ceramide degradation, stimulates accumulation of ceramide, preventing tumor growth [52,53]. SPHK1 inhibitors can also cause tumor cell cytotoxicity, such as dimethylsphingosine [54] and safingol, which concluded phase I clinical trials [55]. Targeting sphingolipid pathway may improve novel strategies for anti-cancer therapeutics, although a few molecules have been tested in clinical trials. Immunotherapy is other growing field that promises significant new advances for cancer therapy [56,57].

## 4. Materials and Methods

### 4.1. Patients and Controls

Peripheral blood was obtained from patients of the Clinics’ Hospital of Federal University of Uberlandia (UFU). All procedures were conducted in accordance with the Declaration of Helsinki and were approved by the UFU Ethics Committee on Human Research (N. 064/30 May 2008). All participants provided formal informed consent before the sample collection. Blood samples were obtained from 8 BC patients, 8 BCC patients and 8 healthy donors (control group), in 5-mL Lithium Heparin Vacutainer tubes (BD vacutainer, Franklin Lakes, NJ, USA). 

### 4.2. Characterization of S1P Receptors by Flow Cytometry

Flow cytometric assays using the fluorescence staining of human cells by lysed whole blood method (Becton Dickinson, Franklin Lakes, NJ, USA) with modifications was performed to determine the presence of S1PRs on lymphocytes, monocytes and granulocytes, simultaneously. Within 1 h, 100 µL of blood was incubated with Pharm Lyse (BD Biosciences, San Jose, CA, USA) for erythrocytes lyse, according to manufacturer’s instructions. For direct assays, cells were labeled with monoclonal antibodies (mAbs) specific for human leukocytes cell-surface markers: anti-CD3 (Alexa Fluor; AF647), anti-CD14 (phycoerythrin; PE), anti-CD16 (AF647), anti-CD66b (peridinin chlorophyll protein cychrome conjugated; PerCP/Cy5.5), all from BioLegend (San Diego, CA, USA). For indirect assays, cells were labeled in the same conditions with the following anti-human antibodies: anti-S1PR1, anti-S1PR2, anti-S1PR3, anti-S1PR4, and anti-S1PR5, all from Sigma-Aldrich (St. Louis, MO, USA), and with fluorescein isothiocyanate-conjugated (FITC) goat anti-mouse lgF (ab) (Life Technologies, Carlsbad, CA, USA) as the secondary antibody. Cells were also labeled with appropriate isotype control antibodies. For each tube, 60.000 events corresponding to viable leukocytes were collected on the Accuri C6 flow cytometer (BD Biosciences, San Jose, CA, USA).

### 4.3. Flow Cytometry Gating Strategy

**Figure 4:**
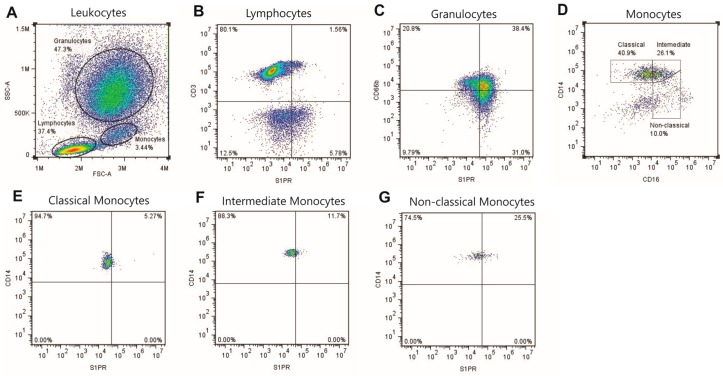
Leukocyte-gating strategy for the measurement of S1PRs in cells subpopulations. Leukocytes were identified in a SSC/FSC plot for granulocytes, lymphocytes, and monocytes selection (**A**); The lymphocyte gate was copied to a CD3/S1PR plot to identify T-cells expressing S1PRs (CD3^+^/S1PR^+^) (**B**); The granulocyte population was copied to a CD66b/S1PR plot identifying granulocytes that present S1PRs (CD66b^+^/S1PR^+^) (**C**); To identify monocytes subsets, monocytes were copied to a CD14/CD16 plot and divided into three populations: classical monocytes (CD14^++^/CD16^−^), intermediate monocytes (CD14^++^/CD16^+^), and non-classical monocytes (CD14^+^/CD16^++^) (**D**); Each monocyte population were copied to a CD14/S1PR plot to evaluate the presence of S1PRs in these monocytes (**E**, **F**, and **G**, respectively).

Subpopulations analyses were accomplished according to distinct gating strategy using the FlowJo software v7.6.5 (Tree Star, San Carlos, CA, USA). An acquisition threshold was set, and unwanted events like platelets, dead cells and debris were not recorded. Quadrants were defined using isotype controls and compensation. First, leukocytes were visualized in a forward scatter (FSC) versus side scatter (SSC) plot, thus three leukocyte subsets were identified: granulocytes, lymphocytes, and monocytes. S1PRs were identified in T lymphocytes from CD3 versus S1PRs plot and in granulocytes from CD66b versus S1PR plot, for each receptor individually. S1PRs in monocytes were defined by sequential gating. First monocytes were classified based on CD14 and CD16 expression in classical (CD14^++^CD16^−^), intermediate (CD14^++^CD16^+^) and non-classical monocytes (CD14^+^CD16^++^), and then S1PRs were identified in each population. Nomenclature of monocytes subsets followed the recommendations of the Nomenclature Committee of the International Union of Immunological Societies [58]. Data were shown as percentage of absolute value of subpopulations (Figure 4).

### 4.4. Gene Expression Analysis for SPHK1, SPHK2, CERK, CERS2, and ACER

RNA from blood was extracted using the TRIzol^®^ LS (Life Technologies, Carlsbad, CA, USA) until phase separation and then proceeded with the aqueous phase using the RNeasy mini kit (Qiagen, Hilden, Germany), both according to the manufacturer’s instructions. The concentration and quality of the RNA were determined by ultraviolet absorbance and electrophoresis. Complementary DNA (cDNA) was generated by reverse transcription (MMLV, Life Technologies, Carlsbad, CA, USA) using 0.5 µg of RNA, according to the manufacturer’s instructions. The qPCR analysis was performed on an ABI PRISM 7300 (Applied Biosystems, Foster City, CA, USA) using the TaqMan Universal PCR Master Mix method for the quantification of *SPHK1*, *SPHK2*, *CERK*, *CERS2*, and *ACER3* in blood samples. The reaction was conducted in a final volume of 12 µL, including 6 µL of master mix (Applied Biosystems, Foster City, CA, USA), 5 µL of cDNA template and 0.2 µL of probe. The following probes were used: *SPHK1*: Hs01116530_g1, *SPHK2*: Hs00219999_m1, CERK: Hs00368483_m1, *ACER3*: Hs00218034_m1, *CERS2*: Hs00371958_g1, and the endogenous control *GAPDH*: Hs03929097_g1 (Applied Biosystems, Foster City, CA, USA). Cycling conditions were 50 °C for 2 min, 95 °C for 10 min, followed by 40 cycles of 95 °C for 15 s and 60 °C for 1 min. All reactions were performed in duplicates. The expression of the target gene transcripts was normalized to glyceraldehyde 3-phosphate dehydrogenase (*GAPDH*), and relative quantification was calculated by 2^−ΔΔ*C*t^ Method, using the control group (healthy donors) as calibrator.

### 4.5. Cytokines Assays

The cytometric bead array (CBA) Human Th1/Th2/Th17 Kit (BD Biosciences, USA) was used to measure blood levels of interleukin IL-2, IL-4, IL-6, IL-10, IL-17A, TNF-α and IFN-γ according to the manufacturer’s instructions. Briefly, samples and human recombinant cytokines (standards curve) were incubated for 3 h at room temperature with a suspension of cytokines capture beads and a phycoerythrin (PE)-conjugated detection reagent. After incubation, the mixture was washed, and measurements were performed on the Accuri C6 flow cytometer (BD Biosciences, San Jose, CA, USA). Cytokine quantification was performed using the FCAP Array Software (v3.0, BD Biosciences, San Jose, CA, USA) to obtain the Median fluorescence intensity (MFI) values. The amount of leukocytes found in complete blood count collected on the same day of the assay was used to normalize cytokine levels.

### 4.6. Statistical Analysis

All statistical data were analyzed using GraphPad Prism 5.0 (GraphPad Software Inc., San Diego, CA, USA). The statistical analysis was performed using one-way analysis of variance (ANOVA), followed by Tukey’s Multiple Comparison test. The correlation analysis was performed using Pearson Correlation coefficient for all statistical data, the level of significance was *p* < 0.05.

## 5. Conclusions

In brief, this study showed that S1PRs are expressed in lymphocytes, monocytes and granulocytes of BC and BCC patients at different levels. The analysis of T lymphocytes revealed that all S1PRs were more expressed in BCC group, whereas in granulocytes the higher levels of these receptors were observed in BC patients. Classical and intermediate monocyte populations presented the lowest receptor levels in both BC and BCC groups, with significant variations found in the non-classical population. The gene expression of the sphingolipid pathway indicated that the pathway is favoring S1P and C1P production in BC, but reversion was observed after chemotherapy treatment. Overall, these data revealed an important role of the sphingolipid pathway in breast cancer immune response, demonstrating that S1PRs presented skewed signaling, which may have favored cancer occurrence and progression. It is important to observe that although chemotherapy has reduced cellular levels of *SPHK1*, an opposite molecular event occurs resulting in higher levels of S1PRs in T lymphocytes, probably increasing the capacity of S1P sensing in the extracellular matrix. Therefore, increasing levels of all five receptors may be part of the initial trigger for drug resistance. It remains to be demonstrated how inhibitors and analogues of key molecules of the S1P signaling pathway will affect immune cells in breast cancer and whether the combination of chemotherapy with such inhibitors may lead to a durable therapeutic response.

## Figures and Tables

**Table 1:** Pearson correlation between *SPHK1* expression and TNF-α and IFN-γ levels in blood samples of breast cancer patients.

Variable	*SPHK1*	
	Pearson Correlation	Sig. (2-Tailed)
TNF-α	0.5287 *	0.0352
IFN-γ	0.4061	0.1185

* Correlation is significant at the 0.05 level (2-tailed). Description: SPHK1: sphingosine kinases 1, TNF-α: Tumor necrosis factor alpha, IFN-γ: Interferon gamma.

## Abbreviations

ACER	ceramidase 3
BC	breast cancer
BCC	breast cancer under chemotherapy treatment
C1P	ceramide 1-phosphate
CERK	ceramide kinase
CERS2	ceramide synthase 2
IDC	invasive ductal carcinoma
ILC	invasive lobular carcinoma
S1P	sphingosine 1-phosphate
SPHK	sphingosine kinase
